# Tocilizumab Therapy and Incident Depression in Rheumatoid Arthritis: A Retrospective Cohort Study of the All of Us Research Program

**DOI:** 10.7759/cureus.113659

**Published:** 2026-07-30

**Authors:** Steven Lehrer, Peter Rheinstein

**Affiliations:** 1 Radiation Oncology, Icahn School of Medicine at Mount Sinai, New York, USA; 2 Family Medicine, Severn Health Solutions, Severna Park, USA

**Keywords:** all of us research program, depression, interleukin-6, rheumatoid arthritis, tocilizumab

## Abstract

Background: Depression is a common and disabling comorbidity in rheumatoid arthritis (RA). Experimental and genetic evidence implicates interleukin-6 (IL-6) signaling in the pathogenesis of depression, but the association between pharmacologic IL-6 receptor inhibition and incident depression in patients with RA remains uncertain.

Methods: We conducted a retrospective cohort study using the National Institutes of Health's All of Us Research Program. Adults with RA and no history of depression before cohort entry were included. Tocilizumab exposure was defined by documented drug administration. The primary outcome was incident depression after the index date. Multivariable logistic regression adjusted for age, sex, race, and ethnicity, inverse probability of treatment weighting (IPTW), and Cox proportional hazards regression were used to estimate the association between tocilizumab exposure and the risk of depression.

Results: The study included 9,826 patients with RA, including 299 treated with tocilizumab and 9,527 untreated controls. Incident depression occurred in 66 of 299 patients (22.1%) treated with tocilizumab and 3,288 of 9,527 patients (34.5%) in the untreated group. Tocilizumab was associated with a lower risk of incident depression in unadjusted analysis (odds ratio (OR), 0.54; 95% confidence interval (CI), 0.42-0.72; P<0.001). The association remained significant after multivariable adjustment (OR, 0.56; 95% CI, 0.42-0.74; P<0.001) and IPTW analysis (OR, 0.58; 95% CI, 0.54-0.61; P<0.001). Cox regression likewise demonstrated a reduced hazard of depression among tocilizumab-treated patients (adjusted hazard ratio (HR), 0.70; 95% CI, 0.55-0.90; P=0.003 by the log-rank test).

Conclusions: Tocilizumab therapy was associated with a significantly lower risk of incident depression among patients with RA. These findings support a potential role for IL-6 receptor blockade in modifying depression risk and warrant prospective studies to determine whether IL-6 inhibition has direct antidepressant effects independent of its effects on inflammatory disease control.

## Introduction

Rheumatoid arthritis (RA) is a chronic autoimmune disease characterized by persistent synovial inflammation, progressive joint destruction, and substantial systemic morbidity [1]. Beyond musculoskeletal manifestations, patients with RA experience a high burden of psychiatric comorbidity, particularly depression, which affects approximately 15%-30% of patients and is associated with reduced quality of life, impaired physical function, poor adherence to disease-modifying therapy, increased healthcare utilization, and premature mortality [2]. Depression itself has emerged as an independent determinant of adverse clinical outcomes in RA, underscoring the importance of identifying modifiable biological pathways that contribute to both inflammatory disease activity and psychiatric symptoms [3,4].

Accumulating evidence supports an important role for inflammation in the pathogenesis of depression [5]. Patients with major depressive disorder exhibit elevated circulating concentrations of pro-inflammatory cytokines, including interleukin-6 (IL-6), tumor necrosis factor-α, and interleukin-1β, while prospective cohort studies have demonstrated that elevated inflammatory biomarkers precede the subsequent development of depressive symptoms [6]. Experimental administration of inflammatory cytokines induces fatigue, anhedonia, cognitive dysfunction, and depressed mood, providing evidence that activation of the innate immune system can directly influence brain function. These observations have led to the inflammatory hypothesis of depression, in which chronic immune activation alters neurotransmitter metabolism, hypothalamic-pituitary-adrenal axis function, neuroplasticity, and microglial activation.

Among inflammatory mediators, IL-6 occupies a particularly important position. IL-6 is abundantly expressed within the inflamed synovium in RA and contributes to leukocyte recruitment, B-cell maturation, T-cell differentiation, osteoclast activation, and hepatic production of acute-phase reactants such as C-reactive protein. Beyond its peripheral immunologic effects, IL-6 signaling influences central nervous system function through multiple mechanisms, including modulation of blood-brain barrier permeability, activation of microglia, alteration of glutamatergic and serotonergic neurotransmission, and impairment of hippocampal neurogenesis. Elevated circulating IL-6 concentrations have repeatedly been associated with depressive symptoms, treatment-resistant depression, and future risk of major depressive disorder, making IL-6 an attractive therapeutic target for inflammation-associated depression [7].

Tocilizumab is a humanized monoclonal antibody directed against both soluble and membrane-bound IL-6 receptors and is an established therapy for moderate-to-severe RA [8]. Clinical trials have consistently demonstrated its efficacy in reducing disease activity, preventing structural joint damage, and improving physical function. However, whether IL-6 receptor blockade also influences depression remains uncertain. Improvements in mood observed during biologic therapy may reflect reduced pain and disability, direct modulation of inflammatory pathways involved in depression, or a combination of both. Previous studies examining psychiatric outcomes during biologic treatment have generally been limited by modest sample sizes, heterogeneous inflammatory diseases, short follow-up, or lack of rigorous adjustment for baseline differences between treated and untreated patients.

Observational studies evaluating medication effects are particularly susceptible to confounding because biologic therapies are prescribed selectively according to disease severity, prior treatment response, comorbidities, and clinician preference. Consequently, analyses relying solely on conventional multivariable adjustment may not adequately account for systematic differences between treatment groups. Contemporary causal inference approaches, including inverse probability of treatment weighting (IPTW) and propensity score matching, provide complementary strategies to improve covariate balance and strengthen causal inference by creating treatment groups that more closely resemble those expected in a randomized clinical trial.

In the present study, we investigated the association between tocilizumab treatment and incident depression among patients with RA in a large real-world cohort. We evaluated this association using conventional multivariable logistic regression and a series of prespecified sensitivity analyses, including restriction to depression occurring after RA diagnosis, IPTW, and propensity score matching. We hypothesized that IL-6 receptor blockade would be associated with a lower likelihood of incident depression and that this association would remain consistent across multiple analytic approaches designed to minimize measured confounding.

The objective of this study was to evaluate the association between tocilizumab therapy and incident depression among adults with RA in the National Institutes of Health's All of Us Research Program. The primary outcome was incident depression after the index date. The primary analysis used multivariable logistic regression adjusted for age, sex, race, and ethnicity, with complementary analyses including IPTW, propensity score matching, and Cox proportional hazards regression to examine the robustness of the observed association. Because this was an observational study, the analyses were designed to evaluate associations rather than establish causality.

## Materials and methods

Study design and data source

We performed a retrospective observational cohort study using electronic health record (EHR) data from the National Institutes of Health's All of Us Research Program. The objective was to evaluate the association between tocilizumab therapy and incident depression among adults with RA. The study was conducted and reported in accordance with the Strengthening the Reporting of Observational Studies in Epidemiology (STROBE) guidelines.

Study population

Patients with RA were identified using diagnostic codes recorded in the EHR. Eligible patients were required to have a documented diagnosis of RA and complete demographic information required for multivariable adjustment. Patients with missing values for age, sex, race, or ethnicity were excluded from the adjusted analyses.

The final analytic cohort consisted of 9,826 patients, including 299 patients exposed to tocilizumab and 9,527 patients not exposed to tocilizumab. The inclusion and exclusion criteria are summarized in Table 1.

**Table 1:** Inclusion/exclusion criteria Eligibility criteria used to define the retrospective cohort of adults with RA from the National Institutes of Health's All of Us Research Program. Patients were required to have a documented diagnosis of RA and sufficient demographic information for multivariable analyses. Patients with a diagnosis of depression before the index date or missing demographic data required for adjusted analyses were excluded. RA: rheumatoid arthritis

Criterion	Description
Inclusion	Adults with RA identified in the All of Us Research Program
Inclusion	Available demographic information (age, sex, race, and ethnicity)
Inclusion	Follow-up after cohort entry
Exclusion	Depression diagnosed before the index date

Exposure

The primary exposure was treatment with tocilizumab, identified from medication records. Patients were classified as exposed if they had at least one documented exposure to tocilizumab during the observation period and unexposed otherwise. Tocilizumab exposure was identified using drug exposure records within the Observational Medical Outcomes Partnership (OMOP) Common Data Model (CDM) of the All of Us Research Program. Because medication adherence, cumulative dose, treatment duration, discontinuation, and persistence were not consistently available, exposure was analyzed as an ever-treated variable. Dose-response relationships and time-varying exposure could not be evaluated.

Outcome

The primary outcome was incident depression identified from OMOP diagnostic concepts recorded in the EHR. Patients with evidence of depression before the index date were excluded. Depression was defined using coded clinical diagnoses rather than standardized psychiatric rating scales or structured psychiatric interviews. Consequently, outcome misclassification is possible, and the diagnosis reflects clinician-recorded depression rather than the severity of depressive symptoms.

To address potential reverse causation, sensitivity analyses were performed by restricting the outcome to depression diagnosed after the diagnosis of RA. An additional analysis excluded patients with evidence of depression before the treatment index date.

Statistical analysis

Covariates included age, sex, race, and ethnicity because these variables were available for nearly all participants and were considered potential confounders of both biologic prescribing and depression risk. Additional clinically relevant variables, including disease activity, corticosteroid exposure, methotrexate use, previous biologic therapy, smoking status, body mass index, socioeconomic status, psychiatric history, and healthcare utilization, were unavailable or incompletely captured and therefore could not be incorporated into the primary analyses.

Baseline characteristics were summarized using descriptive statistics. Continuous variables were presented as means with standard deviations, whereas categorical variables were presented as counts and percentages.

The primary analysis used multivariable logistic regression to estimate the association between tocilizumab exposure and incident depression after adjustment for age, sex, race, and ethnicity.

Prespecified sensitivity analyses included age-adjusted logistic regression, restriction to depression occurring after RA diagnosis, exclusion of depression before treatment initiation, IPTW, propensity score matching, and Cox proportional hazards regression.

Associations were presented as odds ratios (ORs), hazard ratios (HRs), absolute risk differences, and relative risks, where appropriate, together with corresponding 95% confidence intervals (CIs).

Propensity Score Estimation

To reduce confounding by indication, a propensity score representing each patient's probability of receiving tocilizumab was estimated using logistic regression. The propensity score model included age (continuous), sex, race, and ethnicity. The estimated propensity score was subsequently used for both IPTW and propensity score matching.

Inverse Probability of Treatment Weighting

IPTW was used to estimate the average treatment effect while retaining the entire study population. Stabilized inverse probability weights were calculated from the estimated propensity scores. To reduce the influence of extreme observations, weights were truncated at the 99th percentile.

Weighted logistic regression models were fitted using quasibinomial regression, and Huber-White sandwich standard errors were calculated to account for the weighting.

Propensity Score Matching

Nearest-neighbor propensity score matching was performed using a 1:5 treated-to-control ratio without replacement. Matching was conducted on the logit of the propensity score. Following matching, balance between treatment groups was evaluated using standardized mean differences (SMDs). Adequate balance was defined a priori as an absolute SMD of less than 0.10 for all measured covariates. The matched cohort was subsequently analyzed using logistic regression with standard errors clustered on matched subclasses to account for the matched study design.

Sensitivity analyses

Three prespecified sensitivity analyses were performed to evaluate the reliability of the primary findings. First, depression was restricted to diagnoses occurring after the diagnosis of RA to reduce the possibility of reverse causation. Second, patients with depression diagnosed before the treatment index date were excluded. Third, causal inference methods, including IPTW and propensity score matching, were applied to evaluate whether the association between tocilizumab and depression remained consistent after improving covariate balance between treated and untreated patients.

Statistical software

All analyses were performed using R (R Foundation for Statistical Computing, Vienna, Austria). Logistic regression models were fitted using the glm function with a binomial link. Propensity score matching was performed using the MatchIt package, covariate balance was assessed using the cobalt package, and variance estimates were calculated using the sandwich and lmtest packages.

## Results

Study population

The study cohort included 9,826 adults with RA and no documented diagnosis of depression before the index date. Of these, 299 (3.0%) had documented exposure to tocilizumab and 9,527 (97.0%) had no recorded exposure.

Incident depression occurred in 66 of 299 patients (22.1%) receiving tocilizumab and 3,288 of 9,527 untreated patients (34.5%). The absolute risk difference was -12.4%, corresponding to a relative risk of 0.64.

Patients treated with tocilizumab were older than untreated patients (mean age, 58.2 vs. 55.3 years; SMD=0.217). Baseline differences in sex, race, and ethnicity were modest (SMDs 0.108-0.160), indicating mild imbalance between treatment groups prior to adjustment (Table 2).

**Table 2:** Baseline characteristics of patients with RA without prior depression, stratified by tocilizumab exposure Baseline demographic characteristics of 9,826 patients with RA without a prior diagnosis of depression, including 299 patients treated with tocilizumab and 9,527 untreated patients. Continuous variables are presented as mean SD, and categorical variables as number (%). SMDs are presented to assess baseline balance, with values >0.10 indicating meaningful imbalance. Tocilizumab-treated patients were modestly older than untreated patients, with smaller differences in sex, race, and ethnicity distributions. These baseline imbalances were addressed in subsequent adjusted analyses. Percentages do not sum to 100% for sex, race, or ethnicity because participants with missing, unknown, declined-to-answer, or other categories are not shown in the table. Therefore, the calculated n values are approximate conversions from the reported percentages. RA: rheumatoid arthritis; SMDs: standardized mean differences; SD: standard deviation

Characteristic	No tocilizumab (n=9,527)	Tocilizumab (n=299)	SMD
Age, mean (SD)	55.3 (14.1)	58.2 (13.1)	0.217
Sex			
Female	7,250 (76.1%)	235 (78.6%)	0.16
Male	2,125 (22.3%)	59 (19.7%)	
Race			
White population	5,516 (57.9%)	181 (60.5%)	0.158
Black or African American population	1,457 (15.3%)	40 (13.4%)	
Asian population	162 (1.7%)	4 (1.3%)	
American Indian or Alaska Native population	171 (1.8%)	3 (1.0%)	
Middle Eastern or North African population	38 (0.4%)	3 (1.0%)	
More than one population	476 (5.0%)	18 (6.0%)	
Ethnicity			
Hispanic or Latino population	1,705 (17.9%)	44 (14.7%)	0.108
Not Hispanic or Latino population	7,545 (79.2%)	244 (81.6%)	

Among the entire cohort, 3,354 patients had a diagnosis of depression, and 6,472 did not. Among patients treated with tocilizumab, 66 (22.1%) had depression compared with 3,288 (34.5%) of untreated patients. The crude distribution of depression according to treatment status suggested a lower prevalence of depression among patients receiving tocilizumab.

Primary analysis

In the unadjusted logistic regression model, tocilizumab treatment was associated with significantly lower odds of depression (OR 0.54, 95% CI 0.41-0.70; P<0.001).

Adjustment for age alone produced nearly identical results (OR 0.57, 95% CI 0.43-0.75; P<0.001), indicating that age differences between treatment groups explained little of the observed association.

The primary multivariable model adjusted for age, sex, race, and ethnicity likewise demonstrated a significant inverse association between tocilizumab treatment and depression (OR 0.56, 95% CI 0.42-0.73; P<0.001). Age was independently associated with depression, with each additional year associated with approximately a 2% reduction in the odds of depression (OR 0.98 per year, P<0.001).

Sensitivity analyses

To examine temporal relationships between exposure and outcome, analyses were restricted to depression diagnosed after the diagnosis of RA. Under this more stringent definition, the association became slightly stronger, with tocilizumab associated with a 48% reduction in the odds of depression (OR 0.52, 95% CI 0.39-0.69; P<0.001).

An additional sensitivity analysis excluding patients with depression before the treatment index date yielded results essentially identical to those of the primary adjusted model (OR 0.56, 95% CI 0.42-0.73; P<0.001), indicating that prevalent depression before treatment initiation was unlikely to explain the findings.

Inverse probability of treatment weighting

To further reduce measured confounding, IPTW was performed using propensity scores estimated from age, sex, race, and ethnicity. Stabilized weights were truncated at the 99th percentile, and weighted logistic regression was performed with standard errors.

The IPTW analysis confirmed the primary findings. Tocilizumab remained significantly associated with lower odds of depression (OR 0.57, 95% CI 0.52-0.62; P<0.001), providing evidence that the observed association remained significant after weighting the study population to improve covariate balance.

Propensity score matching

Nearest-neighbor propensity score matching without replacement was performed using a 1:5 treated-to-control ratio. The matched cohort included all 299 patients treated with tocilizumab and 1,495 matched untreated controls, while 8,032 untreated patients remained unmatched.

Covariate balance

Prior to matching, treated patients were modestly older than untreated patients (SMD=0.226), and the propensity score itself differed between groups (SMD=0.183), indicating measurable baseline imbalance.

Following matching, balance improved substantially. The SMD for age decreased to -0.015, and the SMD for the propensity score decreased to 0.026. All measured covariates achieved absolute SMDs below the prespecified threshold of 0.10, indicating excellent covariate balance between the treatment groups.

Matched analysis

Within the propensity score-matched cohort, tocilizumab remained significantly associated with lower odds of depression (OR, 0.56; 95% CI, 0.42-0.75; P<0.001). Across all prespecified analyses, the observed association between tocilizumab exposure and incident depression remained similar in magnitude. Although the consistency of these estimates supports the robustness of the observed association across several statistical approaches, all models were adjusted only for available demographic covariates. Therefore, residual confounding cannot be excluded (Table 3).

**Table 3:** Summary of findings Across all prespecified analyses, the estimated association between tocilizumab treatment and incident depression was highly consistent (Table 2). Estimated ORs ranged from 0.52 to 0.57, corresponding to an approximately 43%-48% reduction in the odds of incident depression among patients receiving tocilizumab. OR: odds ratio; RA: rheumatoid arthritis; IPTW: inverse probability of treatment weighting

Analysis	OR	95% CI	P-value
Crude	0.54	0.41-0.70	<0.001
Age-adjusted	0.57	0.43-0.75	<0.001
Demographic-adjusted	0.56	0.42-0.73	<0.001
Depression after RA diagnosis	0.52	0.39-0.69	<0.001
IPTW	0.57	0.52-0.62	<0.001
Propensity score matched	0.56	0.42-0.75	<0.001

The risk of incident depression was 22.1% (66/299) among patients treated with tocilizumab compared with 34.5% (3,288/9,527) among untreated patients. This corresponded to an absolute risk difference of −12.4% and a relative risk of 0.64. These findings indicate that tocilizumab exposure was associated with a lower incidence of depression in this cohort. Because this was an observational study, these results should be interpreted as indicating an association rather than a causal treatment effect.

**Figure 1:**
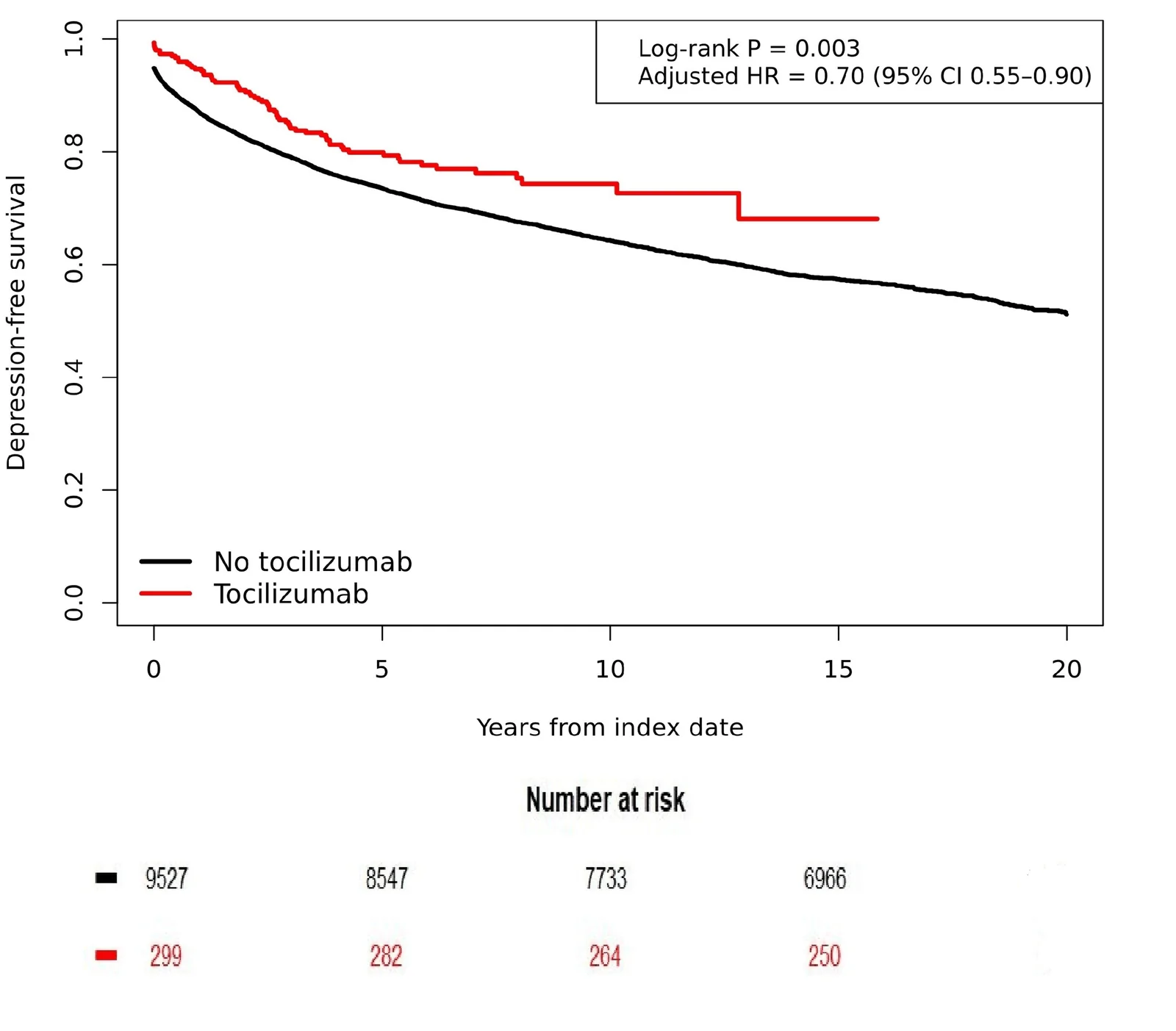
Kaplan-Meier estimates of depression-free survival according to tocilizumab exposure Kaplan-Meier curves showing depression-free survival among 9,826 patients with RA without a prior diagnosis of depression, stratified by tocilizumab exposure (299 treated and 9,527 untreated patients). The index date was defined as the first tocilizumab exposure for treated patients and the RA diagnosis date for untreated patients. Patients receiving tocilizumab had a significantly higher probability of remaining free of incident depression during follow-up than untreated patients (log-rank P=0.003). In a multivariable Cox proportional hazards model adjusted for age, sex, race, and ethnicity, tocilizumab use remained independently associated with a lower risk of incident depression (adjusted HR, 0.70; 95% CI, 0.55-0.90). HR: hazard ratio; CI: confidence interval; RA: rheumatoid arthritis

The close agreement among conventional regression, temporal sensitivity analyses, IPTW, and propensity score matching indicates that the association between tocilizumab treatment and lower odds of depression was significant across multiple statistical methods designed to address measured confounding (Figure 1).

## Discussion

In this retrospective observational cohort study, documented exposure to tocilizumab was consistently associated with a lower incidence of depression among patients with RA. Similar estimates were observed across conventional multivariable regression, IPTW, propensity score matching, and Cox proportional hazards regression. Because of the observational design, these findings should be interpreted as demonstrating an association rather than causation [9].

Depression is among the most clinically important comorbidities of RA, adversely affecting quality of life, functional status, medication adherence, healthcare utilization, and survival. Although psychosocial factors and chronic pain undoubtedly contribute to depression in RA, increasing evidence supports a central role for systemic inflammation in its pathogenesis. IL-6 occupies a particularly important position within this inflammatory network because it contributes both to synovial inflammation and to neuroimmune pathways implicated in depression. Experimental studies have demonstrated that IL-6 influences blood-brain barrier permeability, microglial activation, monoaminergic neurotransmission, hypothalamic-pituitary-adrenal axis regulation, and kynurenine metabolism, providing biologic plausibility for a direct relationship between IL-6 signaling and depressive illness [10].

An important strength of the present study is the remarkable consistency of the estimated treatment effect across multiple complementary analytic approaches. Restricting the analysis to depression diagnosed after RA diagnosis reduced concerns regarding reverse causation. IPTW and propensity score matching produced effect estimates closely resembling those obtained from conventional regression despite using different strategies to address measured confounding. Following propensity score matching, all measured baseline covariates achieved SMDs below the prespecified threshold of 0.10, indicating excellent balance between treatment groups. Although these methods cannot eliminate unmeasured confounding, the close agreement among the analyses argues against the findings being an artifact of any individual statistical technique.

Our findings complement emerging clinical evidence supporting IL-6 inhibition as a potential therapeutic strategy for inflammation-associated depression. Foley and colleagues recently reported encouraging improvements in depressive symptoms among patients receiving IL-6 receptor blockade in a proof-of-concept randomized clinical trial [11]. Although that study was limited by a modest sample size and short follow-up, it provided mechanistic evidence that IL-6 inhibition may improve depressive symptoms. The present study extends those observations by demonstrating an association between tocilizumab exposure and a lower incidence of clinically diagnosed depression in a large real-world population of patients with RA. Together, these studies provide complementary evidence linking IL-6 signaling to depression across both experimental and observational settings.

The principal strength of this study is the use of a large, diverse United States research cohort with standardized EHR data. The association between tocilizumab exposure and incident depression remained consistent across multiple complementary statistical approaches. Nevertheless, these analyses adjusted only for demographic characteristics available within the study dataset and should not be interpreted as eliminating confounding by indication or other sources of residual confounding.

This study has several important limitations. First, because of its retrospective observational design, causal inference cannot be established. Second, important clinical variables that influence both tocilizumab prescribing and depression risk, including RA disease activity, disease duration, corticosteroid exposure, methotrexate use, prior biologic therapy, body mass index, smoking status, socioeconomic status, psychiatric comorbidity, and healthcare utilization, were unavailable or incompletely captured. Third, depression was identified from EHR diagnostic codes rather than standardized psychiatric assessments. Fourth, medication exposure reflected documented drug exposure rather than adherence or cumulative treatment duration. Fifth, because the index date differed between treatment groups (first documented tocilizumab exposure for treated patients and RA diagnosis for untreated patients), immortal time bias cannot be excluded and may have influenced the observed association. Future studies should employ new-user active-comparator designs or time-dependent exposure analyses to address this limitation. Finally, because the comparator group included patients receiving a variety of therapeutic strategies rather than another biologic agent, the findings should be considered hypothesis-generating and require confirmation in studies using active comparators [12-15].

Despite these limitations, the consistency of the findings across conventional regression, temporal sensitivity analyses, IPTW, propensity score matching, and time-to-event analysis provides epidemiologic support for further investigation of IL-6 receptor blockade as a strategy for preventing depression in chronic inflammatory disease. Prospective randomized trials incorporating standardized psychiatric assessments, inflammatory biomarkers, and longitudinal follow-up will be required to determine whether the observed association reflects a direct neuroimmune effect of IL-6 inhibition or improved mood secondary to more effective control of RA.

## Conclusions

In this retrospective cohort study of adults with RA, documented tocilizumab exposure was associated with a lower incidence of depression. Although the association remained consistent across several complementary statistical approaches, the observational design, potential immortal time bias, and residual confounding preclude causal inference. These findings support further investigation of IL-6 receptor blockade through prospective studies specifically designed to evaluate depression outcomes.
